# Fragility of Randomized Clinical Trials Using Mesh in Abdominal Wall Reconstruction

**DOI:** 10.1001/jamanetworkopen.2023.47534

**Published:** 2023-12-13

**Authors:** Sullivan A. Ayuso, Alexis M. Holland, William R. Lorenz, Gregory T. Scarola, John P. Fischer, Neil J. Smart, B. Todd Heniford

**Affiliations:** 1 Division of Gastrointestinal and Minimally Invasive Surgery, Department of Surgery, Carolinas Medical Center, Charlotte, North Carolina; 2 Division of Plastic Surgery, Department of Surgery, Perelman School of Medicine, Philadelphia, Pennsylvania; 3 Department of Colorectal Surgery, Royal Devon and Exeter NHS Foundation Trust, Royal Devon and Exeter Hospital, Exeter, Devon, England

## Abstract

This systematic review evaluates the fragility of randomized clinical trials that used mesh in abdominal wall reconstruction.

## Introduction

The fragility index (FI) was developed to assess the robustness of clinical trials. The FI calculates the number of individual outcomes that would need to change to make a significant result insignificant using the Fischer exact test.^1^ The FI will prevent reliance solely on the *P* value and help augment other quality metrics used to evaluate randomized clinical trials (RCTs). If a study is significant and has a low FI, it may call into question the strength of the study. The reverse fragility index (RFI) is the inverse of the FI and is meant for trials that report an insignificant finding. Previous systematic reviews have suggested that studies with an FI or RFI score of 3 or less are considered fragile.^2^ The fragility quotient (FQ) is another quantifiable measurement of robustness, where FI accounts for sample size. A larger FQ indicates more robust study outcomes.^3^ The field of abdominal wall reconstruction (AWR) has evolved from single-institution retrospective studies to multicenter RCTs.^4^ This study aims to evaluate the fragility of RCTs that used mesh in AWR.

## Methods

This systematic review followed the Preferred Reporting Items for Systematic Reviews and Meta-analyses (PRISMA) reporting guideline. Informed consent was not required for this study because data were publicly available.

Google Scholar and PubMed were queried for RCTs that used mesh in AWR from 2000 to 2023 (eAppendix in Supplement 1). The first 200 studies from each search engine were reviewed along with their cited references. RCTs needed to have a dichotomous and clinical primary outcome because fragility does not apply to continuous outcomes. Only studies evaluating open ventral and incisional hernias were included; parastomal hernias were excluded. RCTs that were included compared postoperative outcomes between mesh and no-mesh repairs, biologic and synthetic mesh repairs, and various open repair techniques. Methods of mesh placement and patient follow-up were often varied within individual studies. The fragility quotient (FQ) and reverse fragility quotient (RFQ) were determined by dividing the FI or RFI by the sample size of the study. The impact factor (IF) of each journal and the conflicts of interest (COI) of each article were also reported.^5^ SAS, version 9.4 (SAS Institute) was used. A 2-sided Fischer exact test was used to calculate *P* values. Significance was set at *P* < .05. Data were analyzed in September 2023.

## Results

**Figure 1.**
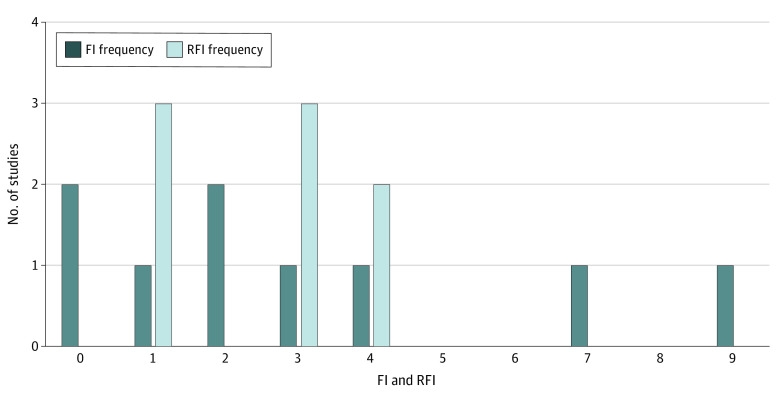
Frequency of Fragility Index (FI) and Reverse Fragility Index (RFI) Values in Mesh Randomized Clinical Trials The frequency of FI and RFI values are displayed for both positive and negative studies.

**Figure 2.**
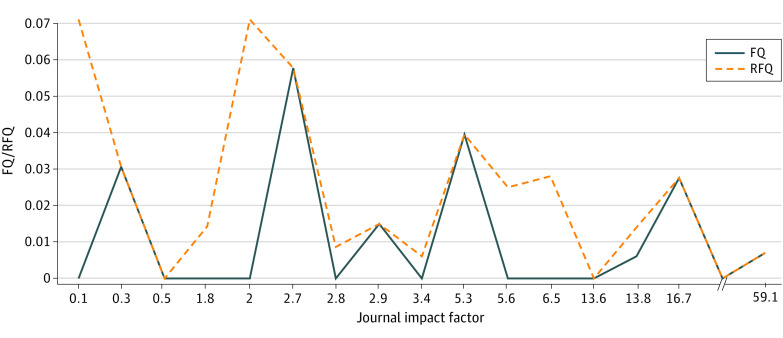
Association of Impact Factor With Randomized Clinical Trial Fragility Impact factor was not associated with the fragility quotient (FQ) or reverse fragility quotient (RFQ).

After exclusion criteria were applied, 17 RCTs were included—9 (52.9%) positive and 8 (47.1%) negative RCTs. The most common journals were the *British Journal of Surgery* (3 [17.6%]), *JAMA Surgery* (2 [11.8%]), and *Annals of Surgery* (2 [11.8%]). Hernia recurrence (7 [41.2%]) and major complications (5 [29.4%]) were the most frequent primary outcomes. The median (range) FI for positive RCTs was 2 (0-9), and the median (range) RFI for negative RCTs was 3 (1-4). There were no significant differences between FIs and RFIs. For all studies, 12 of 17 (70.6%) had an FI or RFI of 3 or less. Four studies had an FI or RFI of 1, and 2 studies had an FI of 0, which indicated that the study outcomes would no longer be significant if a different statistical test was used for the same data (Figure 1). The median (IQR) FQ was 0.015 (0.007-0.031), and the median (IQR) RFQ was 0.020 (0.008-0.039). The journal IF was not associated with FI or RFI (Figure 2). The study that was published in the journal with the highest IF (IF, 59.1) had an FI of 2. The COI was reported in 5 studies (29.4%) and 4 of these studies (80.0%) had an FI or RFI of 3 or less.

## Discussion

The findings of our study suggest that RCTs that evaluated mesh use were fragile and lacked robustness, which is concerning because a change in a small number of patient outcomes would alter the conclusions of fragile studies. Ventral hernia repair is one of the most common operations; however, few RCTs evaluate mesh use during these repairs. The goal of the FI and FQ is to provide another framework for readers to critically and comprehensively evaluate research. However, a limitation to the fragility analysis in hernia repairs is that only binary data can be assessed, while continuous outcomes, such as pain, cost, and operating time, are commonly investigated. Study fragility should be strongly considered when interpreting RCTs, especially when their results may change clinical practice.

## Notes

Supplement 1.
eAppendix.

eReferences.

Supplement 2.
Data Sharing Statement
