# Supplementary material for: Fragility of Randomized Clinical Trials Using Mesh in Abdominal Wall Reconstruction
Source: JAMA Netw Open. 2023 Dec 13;6(12):e2347534. doi: 10.1001/jamanetworkopen.2023.47534 (PMC10719754; doi:10.1001/jamanetworkopen.2023.47534)
Supplement: Supplement 1. — eAppendix. eReferences. [file jamanetwopen-e2347534-s001.pdf]

Supplemental Online Content

Ayuso SA, Holland AM, Lorenz WR, et al. Fragility of randomized clinical trials using mesh in abdominal wall reconstruction. *JAMA Netw Open*. 2023;6(12):e2347534. doi:10.1001/jamanetworkopen.2023.47534

eAppendix. Study Inclusion Criteria

eReferences.

This supplemental material has been provided by the authors to give readers additional information about their work.

© 2023 Ayuso SA et al. *JAMA Netw Open.*

Appendix – Study Inclusion Criteria

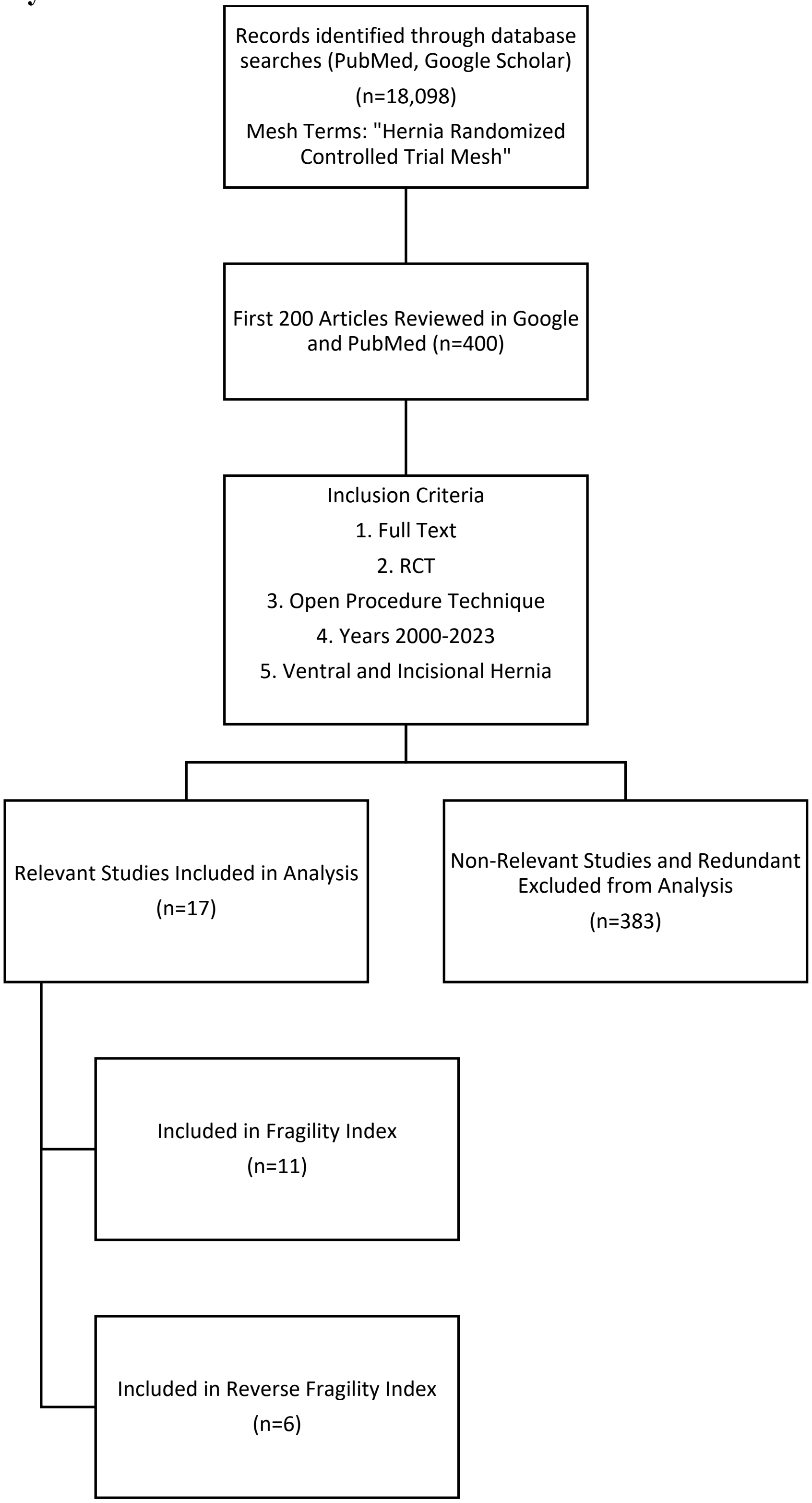

© 2023 Ayuso SA et al. *JAMA Netw Open.*

Included Studies

1. Coelho R, Dhanani NH, Lyons NB, et al. Hernia Prevention Using Biologic Mesh and/or Small Bites: A Multi-Specialty 2x2 Factorial Randomized Controlled Trial. *J Am Coll Surg*. 2023;Publish Ah:309-317. doi:10.1097/xcs.0000000000000705
2. Abdel-Baki NA, Bessa SS, Abdel-Razek AH. Comparison of prosthetic mesh repair and tissue repair in the emergency management of incarcerated para-umbilical hernia: A prospective randomized study. *Hernia*. 2007;11(2):163-167. doi:10.1007/s10029-007-0189-4
3. Ulutas ME, Sahin A, Simsek G, et al. Does onlay mesh placement in emergency laparotomy prevent incisional hernia? A prospective randomized double-blind study. *Hernia*. 2023;27(4):883-893. doi:10.1007/s10029-023-02770-7
4. Demetrashvili Z, Pipia I, Loladze D, et al. Open retromuscular mesh repair versus onlay technique of incisional hernia: A randomized controlled trial. *Int J Surg*. 2017;37:65-70. doi:10.1016/j.ijsu.2016.12.008
5. Sevinç B, Okuş A, Ay S, Aksoy N, Karahan Ö. Randomized prospective comparison of long-term results of onlay and sublay mesh repair techniques for incisional hernia. *Turkish J Surg*. 2018;34(1):17-20. doi:10.5152/turkjsurg.2017.3712
6. Ahmed M, Mehboob M. Comparisons of onlay versus sublay mesh fixation technique in ventral abdominal wall incisional hernia repair. *J Coll Physicians Surg Pakistan*. 2019;29(9):819-822. doi:10.29271/jcpsp.2019.09.819
7. Harris HW, Primus F, Young C, et al. Preventing Recurrence in Clean and Contaminated Hernias Using Biologic Versus Synthetic Mesh in Ventral Hernia Repair: The PRICE Randomized Clinical Trial. *Ann Surg*. 2021;273(4):648-655. doi:10.1097/SLA.0000000000004336
8. Miserez M, Lefering R, Famiglietti F, et al. Synthetic versus biological mesh in laparoscopic and open ventral hernia repair (LAPSIS): Results of a multinational, randomized, controlled, and double-blind trial. *Ann Surg*. 2021;273(1):57-65. doi:10.1097/SLA.0000000000004062
9. Olavarria OA, Bernardi K, Dhanani NH, et al. Synthetic versus Biologic Mesh for Complex Open Ventral Hernia Repair: A Pilot Randomized Controlled Trial. *Surg Infect (Larchmt)*. 2021;22(5):496-503. doi:10.1089/sur.2020.166
10. Rosen MJ, Krpata DM, Petro CC, et al. Biologic vs Synthetic Mesh for Single-stage Repair of Contaminated Ventral Hernias: A Randomized Clinical Trial. *JAMA Surg*. 2022;157(4):293-301. doi:10.1001/jamasurg.2021.6902
11. Arroyo A, Âa PG, Pe Â Rez F, Andreu J, Candela F, Calpena R. *Randomized Clinical Trial Comparing Suture and Mesh Repair of Umbilical Hernia in Adults*. www.bjs.co.uk
12. Kaufmann R, Halm JA, Eker HH, et al. Mesh versus suture repair of umbilical hernia in adults: a randomised, double-blind, controlled, multicentre trial. *Lancet*. 2018;391(10123):860-869. doi:10.1016/S0140-6736(18)30298-8
13. Korenkov M, Sauerland S, Arndt M, Bograd L, Neugebauer EAM, Troidl H. Randomized clinical trial of suture repair, polypropylene mesh or autodermal hernioplasty for incisional hernia. *Br J Surg*. 2002;89(1):50-56. doi:10.1046/j.0007-1323.2001.01974.x
14. Conze J, Kingsnorth AN, Flament JB, et al. Randomized clinical trial comparing lightweight composite mesh with polyester or polypropylene mesh for incisional hernia repair. *Br J Surg*. 2005;92(12):1488-1493. doi:10.1002/bjs.5208

© 2023 Ayuso SA et al. *JAMA Netw Open.*

15. Ponten JEH, Leenders BJM, Leclercq WKG, et al. Mesh Versus Patch Repair for Epigastric and Umbilical Hernia (MORPHEUS Trial); One-Year Results of a Randomized Controlled Trial. *World J Surg*. 2018;42(5):1312-1320. doi:10.1007/s00268-017-4297-8
16. Rabie M, Abdelnaby M, Morshed M, Shalaby M. Posterior component separation with transversus abdominis muscle release versus mesh-only repair in the treatment of complex ventral-wall hernia: a randomized controlled trial. *BMC Surg*. 2022;22(1):1-12. doi:10.1186/s12893-022-01794-7
17. Kohler A, Lavanchy JL, Lenoir U, Kurmann A, Candinas D, Beldi G. Effectiveness of Prophylactic Intraperitoneal Mesh Implantation for Prevention of Incisional Hernia in Patients Undergoing Open Abdominal Surgery: A Randomized Clinical Trial. *JAMA Surg*. 2019;154(2):150-158. doi:10.1001/jamasurg.2018.4221

© 2023 Ayuso SA et al. *JAMA Netw Open.*

Table of Studies Included

Title	Authors	Publication Year	N	Primary Outcome
Comparison of Prosthetic Mesh repair and Tissue Repair in the Emergency Management of Incarcerated Para-Umbilical Hernia: A Prospective Randomized Study	Abdel-Baki et al	2023	40	Postoperative Complications
Comparisons of Onlay Versus Sublay Mesh Fixation Technique in Ventral Abdominal Wall Incisional Hernia Repair	Ahmed et al	2019	65	Seroma Formation
Randomized Clinical Trial Comparing Suture and Mesh Repair of Umbilical Hernia in Adults	Arroyo et al	2001	200	Recurrence
Hernia Prevention Using Biologic Mesh and/or Small Bites: A Multi-Specialty 2x2 Factorial Randomized Controlled Trial	Coelho et al	2023	107	Major Complications
Randomized Clinical Trial Comparing Lightweight Composite Mesh with Polyester or Polypropylene Mesh for Incisional Hernia Repair	Conze et al	2005	165	Recurrence
Open Retromuscular Mesh Repair Versus Onlay Technique of Incisional Hernia: A Randomized Controlled Trial	Demetrashvii et al	2017	155	Wound Complications
Preventing Recurrence in Clean and Contaminated Hernias Using Biologic Versus Synthetic Mesh in Ventral Hernia Repair: The PRICE Randomized Clinical Trial	Harris et al	2021	165	Recurrence
Mesh Versus Suture Repair of Umbilical Hernia in Adults: a Randomised, Double-blind, Controlled, Multicentre Trial	Kaufmann et al	2018	300	Recurrence
Effectiveness of Prophylactic Intraperitoneal Mesh Implantation for Prevention of Incisional Hernia in Patients Undergoing Open Abdominal Surgery: A Randomized Clinical Trial	Kohler et al	2019	150	Incisional Hernia Incidence
Randomized Clinical Trial of Suture Repair, Polypropylene Mesh or Autodermal Hernioplasty for Incisional Hernia	Korenkov et al	2002	160	Recurrence
Synthetic Versus Biological Mesh in Laparoscopic and Open Ventral Hernia Repair (LAPSIS): Results of a Multinational, Randomized, Controlled, and Double-blind Trial	Miserez et al	2021	127	Major Complications
Synthetic versus Biologic Mesh for Complex Open Ventral Hernia Repair: A Pilot Randomized Controlled Trial	Olavarria et al	2021	87	Major Complications

© 2023 Ayuso SA et al. *JAMA Netw Open.*

Mesh Versus Patch Repair for Epigastric and Umbilical Hernia (MORPHEUS Trial); One-Year Results of a Randomized Controlled Trial	Ponten et al	2017	352	Postoperative Complications
Posterior Component Separation with Transversus Abdominis Muscle Release versus Mesh-only Repair in the Treatment of Complex Ventral-wall Hernia: a Randomized Controlled Trial	Rabie et al	2022	56	Recurrence
Biologic vs Synthetic Mesh for Single-stage Repair of Contaminated Ventral Hernias: A Randomized Clinical Trial	Rosen et al	2022	253	Recurrence
Randomized Prospective Comparison of Long-term Results of Onlay and Sublay Mesh Repair Techniques for Incisional Hernia	Sevinc et al	2018	100	Wound Complications
Does Onlay Mesh Placement in Emergency Laparotomy Prevent Incisional Hernia? A Prospective Randomized Double-blind Study	Ulutas et al	2023	108	Incisional Hernia Incidence

© 2023 Ayuso SA et al. *JAMA Netw Open.*
