# Supplementary material for: Fragility of Randomized Clinical Trials Using Mesh in Abdominal Wall Reconstruction
Source: JAMA Netw Open. 2023 Dec 13;6(12):e2347534. doi: 10.1001/jamanetworkopen.2023.47534 (PMC10719754; doi:10.1001/jamanetworkopen.2023.47534)
Supplement: Supplement 2. — Data Sharing Statement [file jamanetwopen-e2347534-s002.pdf]

Data Sharing Statement

Ayuso. Fragility of Randomized Clinical Trials Using Mesh in Abdominal Wall Reconstruction. *JAMA Netw Open*. Published December 13, 2023. doi:10.1001/jamanetworkopen.2023.47534

Data
Data available: Yes
Data types: Data (not involving human participants), Data dictionary
How to access data: Data will be sent by an individual - Todd.Heniford@gmail.com
When available: With publication

Supporting Documents
Document types: None

Additional Information
Who can access the data: Anyone requesting the data
Types of analyses: Fragility Score
Mechanisms of data availability: with investigator support
Any additional restrictions: None
